# Chelerythrine enhances anti-fungi immunity in *Caenorhabditis elegans* via DAF-16 and NHR-49 mediated fatty acid metabolism

**DOI:** 10.3389/fimmu.2026.1828802

**Published:** 2026-06-09

**Authors:** Shenyuan Fan, Guohui Bai, Tingting Zhong, Yi Xiao, Yuan Tian

**Affiliations:** 1 Key Laboratory of Oral Disease Research of the Education Department of Guizhou Province, School of Stomatology, Zunyi Medical University, Zunyi, Guizhou, China; 2 The Key Laboratory of Pharmaceutical Research for Tumor Prevention and Treatment of the Education Department of Guizhou Province, Zunyi Medical University, Zunyi, Guizhou, China; 3 College of Basic Medicine, Zunyi Medical University, Zunyi, Guizhou, China; 4 Institute of Life Sciences, Zunyi Medical University, Zunyi, Guizhou, China

**Keywords:** *Caenorhabditis elegans*, Chelerythrine, DAF-16/FOXO, innate immunity, NHR-49

## Abstract

Chelerythrine is a natural benzophenanthridine alkaloid with various pharmacological activities. However, whether Chelerythrine can influence innate immunity and its underlying molecular mechanisms remain unclear. In this study, we found that 10 μM Chelerythrine significantly extended the lifespan of *Caenorhabditis elegans* infected with *Candida albicans* (*C. albicans*) and inhibited the proliferation of *C. albicans*. This enhanced host resistance to infection was not achieved by reducing the intestinal fungal burden. Transcriptomic sequencing analysis revealed that Chelerythrine activates the FoxO and Fatty acid metabolism pathways in *C. elegans*. Interestingly, the lifespan-extending effect of Chelerythrine was completely abolished in *daf-16* and *nhr-49* mutants. Similarly, mutations in the fatty acid desaturase genes *fat-5*, *fat-6*, and *fat-7* also blocked this protective effect. RT-qPCR results confirmed that Chelerythrine treatment significantly upregulated the expression of FoxO pathway downstream genes (*sod-3*, *thn-2*, *lys-7*) and fatty acid metabolism-related genes (*nhr-49*, *mdt-15*, *fat-5*, *fat-6*, *fat-7*). Fluorescent reporter gene assays further demonstrated that Chelerythrine promotes the nuclear localization of DAF-16::GFP and enhances the fluorescence expression of SOD-3::GFP, FAT-5::GFP, FAT-6::GFP, and FAT-7::GFP. Additionally, broad-spectrum antibacterial assays showed that 10 μM Chelerythrine had no direct inhibitory activity against various pathogens, including *Listeria monocytogenes*, *Enterococcus faecalis*, *Pseudomonas aeruginosa*, and *Salmonella enterica*, indicating that it does not enhance host immunity by directly suppressing pathogen growth. In summary, this study demonstrates that Chelerythrine enhances the innate immune response of *C. elegans* against *C. albicans* by activating the DAF-16/FoxO pathway and the NHR-49-mediated fatty acid metabolism pathway. Our work reveal that Chelerythrine is a potential therapeutic candidate for the treatment of *C. albicans* infections.

## 1 Introduction

Candidiasis, primarily caused by *Candida albicans*, is a globally prevalent opportunistic infection (1, 2). This disease has a significantly higher incidence in immunocompromised populations, such as neonates, the elderly, and HIV-infected individuals, and can lead to mucosal damage, feeding difficulties, and even severe systemic infections (3). Currently, azole drugs remain the first-line clinical therapy. However, due to factors such as the long-term use of antifungal agents, the formation of *C. albicans* biofilms, and genetic mutations, the resistance of *C. albicans* to existing antifungal drugs continues to rise, making the development of new prevention and treatment strategies particularly urgent (4, 5). The World Health Organization (WHO) recently released its first fungal priority pathogen list, based on concerns regarding “ concerns over drug resistance and/or treatment management,” in which *C. albicans* was listed as one of the four “critical priority pathogens” (6). In this context, beyond directly inhibiting the pathogenic microorganisms, enhancing host innate immunity through pharmacological intervention to proactively boost defensive capacity has emerged as a promising complementary therapeutic strategy (7). Therefore, exploring natural products with immunomodulatory activity from traditional medicinal plants provides an important avenue for discovering novel anti-infective agents.

Chelerythrine (CHE) is a natural benzophenanthridine alkaloid extracted from plants such as *Chelidonium majus* L. of the *Papaveraceae* family (8). Studies have demonstrated that Chelerythrine exhibits broad-spectrum biological activities, including antifungal, anti-inflammatory, anticancer, and antiviral properties (9–13). However, whether Chelerythrine can enhance host resistance against *Candida albicans* by modulating innate immune function and the underlying mechanisms involved remain to be elucidated. To elucidate the molecular mechanisms underlying drug-mediated modulation of innate immunity, the selection of an appropriate research model is essential. The nematode *Caenorhabditis elegans* serves as a well-established model organism, characterized by its small size, short life cycle, and well-defined genetic background. Its innate immune defense system is highly conserved throughout evolution, making it widely utilized in studies of host-pathogen interactions and drug activity screening (14). The validity of this model in fungal infection research has been thoroughly established, including its application in studies involving *Candida albicans* infection (15, 16).

DAF-16 is a critical FoxO family transcription factor in *C. elegans* that is primarily regulated by the insulin/insulin-like growth factor-1 signaling pathway (17). The DAF-16 insulin-like signaling pathway is evolutionarily conserved and governs multiple aspects of organismal physiology, including pathogen resistance, metabolism, stress response, and longevity. Upon exposure to stresses such as pathogenic infection, DAF-16 is activated and translocates to the nucleus, where it initiates the expression of a suite of target genes involved in diverse processes including antioxidant defense (e.g., *sod-3*), antimicrobial immunity (e.g., *lys-7*) *(*17, 18), autophagy, and metabolic reprogramming, thereby systemically enhancing organismal survival and immune defense. On the other hand, NHR-49, the functional ortholog of mammalian peroxisome proliferator-activated receptor alpha (PPARα), serves as a master regulator of lipid metabolism in *C. elegans (*19). It maintains lipid homeostasis by modulating fatty acid β-oxidation, the expression of desaturases (e.g., *fat-5*, *fat-6*, *fat-7*), and lipid droplet dynamics (17). Accumulating evidence indicates that lipid metabolites not only function as energy sources but also act as critical mediators of immune signal transduction. NHR-49-mediated lipid remodeling is indispensable for effective resistance against pathogenic infection. DAF-16 and NHR-49 pathways engage in extensive crosstalk, forming an integrated immune-metabolic regulatory network. However, the precise mechanisms by which specific pharmacological agents, such as Chelerythrine, modulate this network to enhance host immunity remain to be elucidated.

In this study, we investigated the role of Chelerythrine in host defenses of *C. elegans*. Via transcriptomic sequencing and GO analysis, we found that Chelerythrine protected host against *Candida albicans* (*C. albicans*) through the Forkhead box O (FoxO) signaling pathway and fatty acid metabolism pathway. In addition, Chelerythrine enhanced the innate immunity through the activation of the transcription factor DAF-16 and the nuclear receptor NHR-49. Given the evolutionary conservation of the FoxO signaling and fatty acid metabolism pathways, these findings suggest that Chelerythrine enhances innate immunity through a mechanism that is likely conserved across species.

## 2 Results

### 2.1 Chelerythrine enhances anti-fungi immunity in *C. elegans*

**Figure 1:**
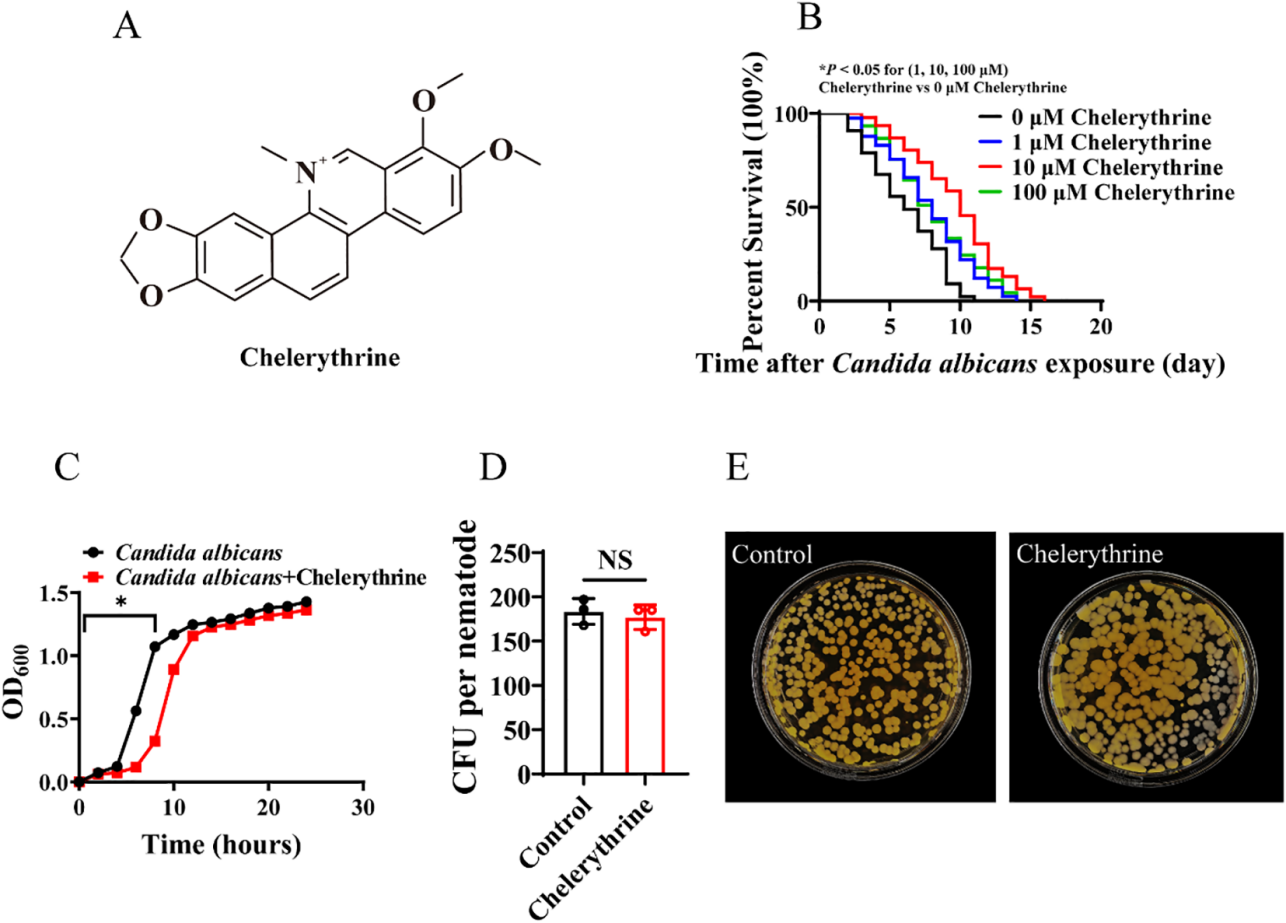
Chelerythrine enhances anti-fungi immunity in *C. elegans*. **(A)** Chelerythrine chemical structure. **(B)** Survival of N2 hermaphrodite worms exposed to increasing concentrations of Chelerythrine following *C. albicans* infection (*P < 0.05, log-rank test; n > 40 per group). **(C)** Chelerythrine (10 μM) significantly inhibits the proliferation of *C. albicans* in liquid culture. Data are presented as mean ± SEM of three independent biological replicates (*P < 0.05, unpaired two-tailed Student’s t-test). **(D)** Chelerythrine (10 μM) did not affect the colony-forming units (CFUs) in the intestines of WT worms after *C. albicans* infection. Data are presented as mean ± SEM of three independent experiments. (n ≥ 20 per group. ns, not significant; unpaired two-tailed Student’s t-test). **(E)** Representative images of SDA agar plates showing *C. albicans* colonies recovered from intestinal lysates of infected worms treated with or without 10 μM Chelerythrine for 48 hours. These results are mean ± SEM of three independent experiments. NS, no significance. (*P < 0.05, unpaired t-test).

To investigate whether Chelerythrine promotes innate immunity, worms were exposed to the human opportunistic pathogen *C. albicans*. The chemical structure of Chelerythrine is shown (**Figure 1A**). We found that wild-type animals treated with Chelerythrine (0 μM, 1 μM, 10 μM, 100 μM) exhibited increased resistance to *C. albicans* in a dose-dependent manner. Meanwhile, Chelerythrine showed a saturating effect on pathogen resistance, with maximal effect at 10 μM and a decline at 100 μM, possibly due to toxicity at high concentrations (**Figure 1B**). These results suggested that Chelerythrine enhances innate immunity in *C. elegans*. To test whether Chelerythrine promotes host immune responses by directly inhibiting the growth of pathogenic fungi, we performed a fungal growth assay. The results showed that 10 μM Chelerythrine significantly inhibited the proliferation of *C. albicans* (**Figure 1C**), suggesting a potential direct antifungal effect. Given that clearance of fungal load is part of host defense against pathogen infection (20, 21), we further examined whether Chelerythrine influenced fungal accumulation *in vivo*. Interestingly, the number of fungal cells in the intestines of Chelerythrine-treated worms did not decrease compared to that in control animals (**Figures 1D, E**). Overall, these results suggest that Chelerythrine protects worms against *C. albicans* infection.

### 2.2 Chelerythrine increases resistance to multiple pathogens

**Figure 2:**
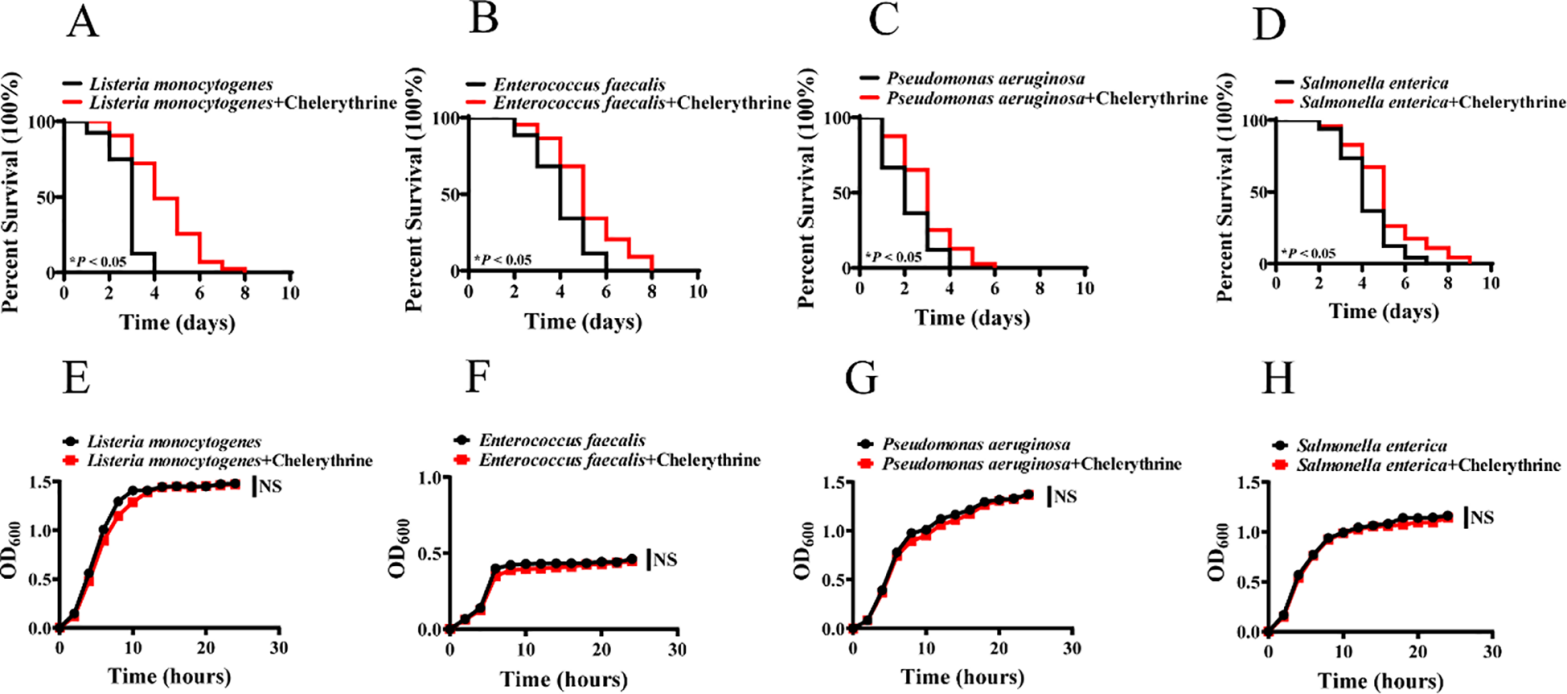
Chelerythrine increases the resistance to pathogens. Chelerythrine (10 μM) enhances the resistance of *C. elegans* to *L. monocytogenes* **(A)**, *E. faecalis* **(B)**, *P. aeruginosa* **(C)** and *S. enterica* **(D)** infection (*P < 0.05, log-rank test; n> 40 per group). Chelerythrine (10 μM) does not suppress the proliferation of *L. monocytogenes* **(E)**, *E. faecalis* **(F)**, *P. aeruginosa* **(G)**, or *S. enterica* **(H)** in liquid culture. Data are presented as mean ± SEM of three independent biological replicates. No significant differences were detected between Chelerythrine-treated and control groups at the 24-hour endpoint (unpaired two-tailed Student’s t-test).

To investigate whether Chelerythrine promotes resistance to other pathogens, we exposed worms to *Listeria monocytogenes*, *Enterococcus faecalis*, *Pseudomonas aeruginosa*, or *Salmonella enterica* in the presence of 10 μM Chelerythrine. Chelerythrine treatment significantly promoted host survival against all tested bacterial pathogens (**Figures 2A–D**), suggesting that Chelerythrine conferred broad-spectrum pathogen resistance. Furthermore, to test whether Chelerythrine enhances host immune responses by directly inhibiting bacterial growth, we performed bacterial growth assays. The results demonstrated that 10 μM Chelerythrine did not suppress the proliferation of *L. monocytogenes*, *E. faecalis*, *P. aeruginosa*, or *S. enterica* (**Figures 2E–H**). Taken together, these results suggest that Chelerythrine increases resistance to multiple pathogens through host immune modulation rather than direct antimicrobial activity.

### 2.3 Transcriptomic sequencing reveals significant enrichment of differentially expressed genes in the Forkhead box O signaling pathway and fatty acid metabolism pathway

**Figure 3:**
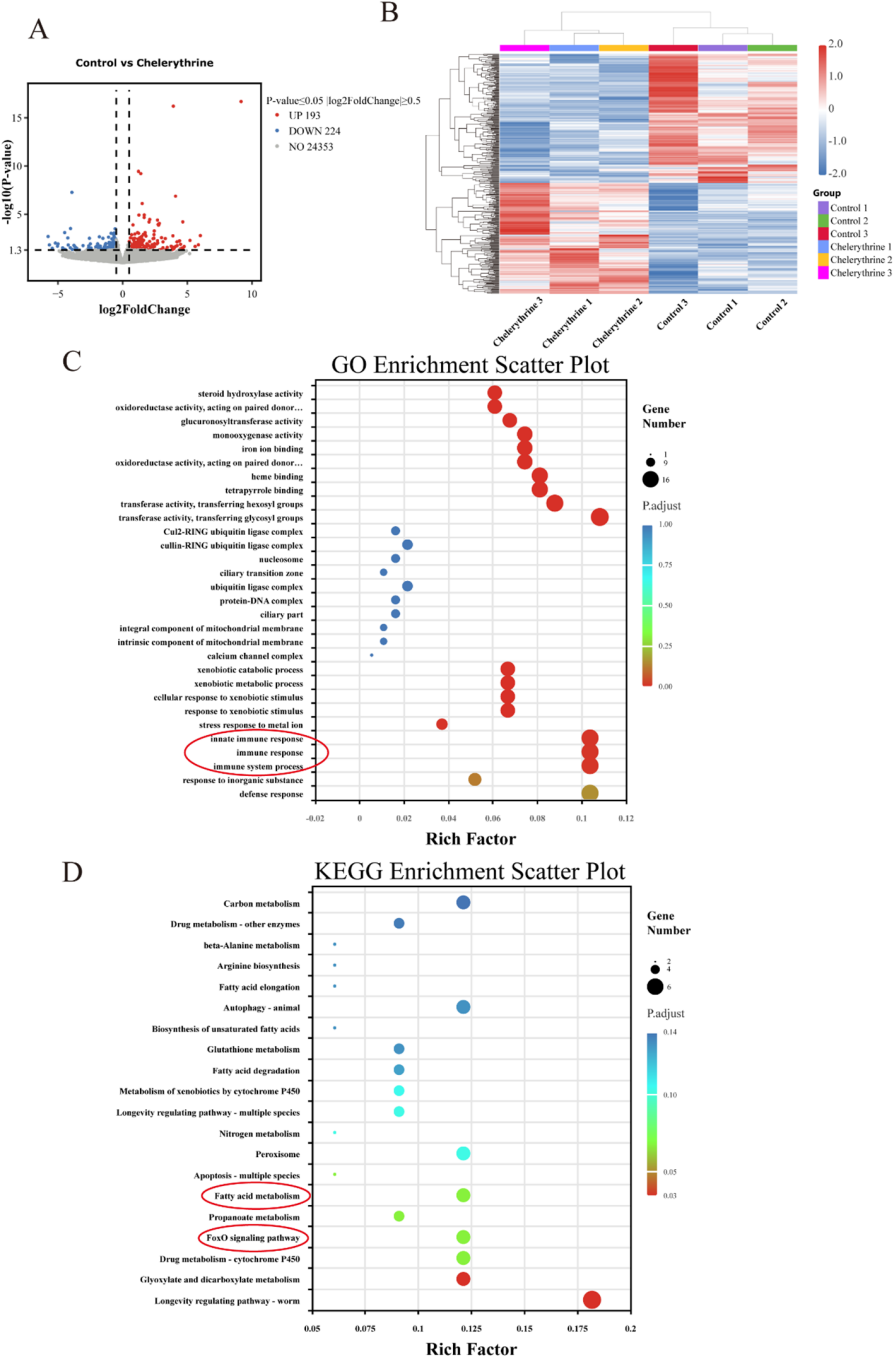
Transcriptomic analysis reveals enrichment of differentially expressed genes (DEGs) in *C. albicans*-infected worms following Chelerythrine treatment. **(A)** Volcano plot showing differentially expressed genes (DEGs) between Chelerythrine-treated and control groups. Genes were considered differentially expressed if FDR < 0.05 and | log_2_FoldChange | ≥ 0.5. Red dots indicate upregulated genes; blue dots indicate downregulated genes. **(B)** Heatmap clustering of DEGs in Chelerythrine-treated worms versus controls. “Chelerythrine 1, 2, 3” and “Control 1, 2, 3” represent three independent biological replicates. **(C)** Gene Ontology (GO) enrichment analysis of DEGs. Enrichment significance was determined using a hypergeometric test with FDR < 0.05. **(D)** Kyoto Encyclopedia of Genes and Genomes (KEGG) pathway enrichment analysis of DEGs. Enrichment significance was determined using a hypergeometric test with FDR < 0.05.

To mechanistically elucidate Chelerythrine’s protective effects, we conducted whole-transcriptome RNA sequencing. Specifically, wild-type N2 worms were infected with C. albicans and simultaneously treated with 10 μM Chelerythrine or vehicle control for 48 hours prior to RNA extraction, thereby capturing transcriptomic changes that occur during active infection. Analysis revealed significant alterations in the nematode transcriptome following Chelerythrine exposure. We identified a total of 417 differentially expressed genes (DEGs), comprising 193 upregulated and 224 downregulated genes. Genes with false discovery rate (FDR) < 0.05 and | log2FoldChange | ≥ 0.5 were considered differentially expressed. (**Figures 3A, B**). The Gene Ontology (GO) enrichment analysis of the DEGs revealed that Chelerythrine treatment primarily affected genes involved in three key immune-related processes: immune response, innate immune response, immune system process (**Figure 3C**). Kyoto Encyclopedia of Genes and Genomes (KEGG) enrichment analysis further showed that the DEGs were significantly enriched in two key pathways: the Forkhead box O (FoxO) signaling pathway and the fatty acid metabolism pathway (**Figure 3D**).

### 2.4 Chelerythrine activates the Forkhead box O transcription factor DAF-16 to enhance anti-fungi immunity in *C. elegans*

**Figure 4:**
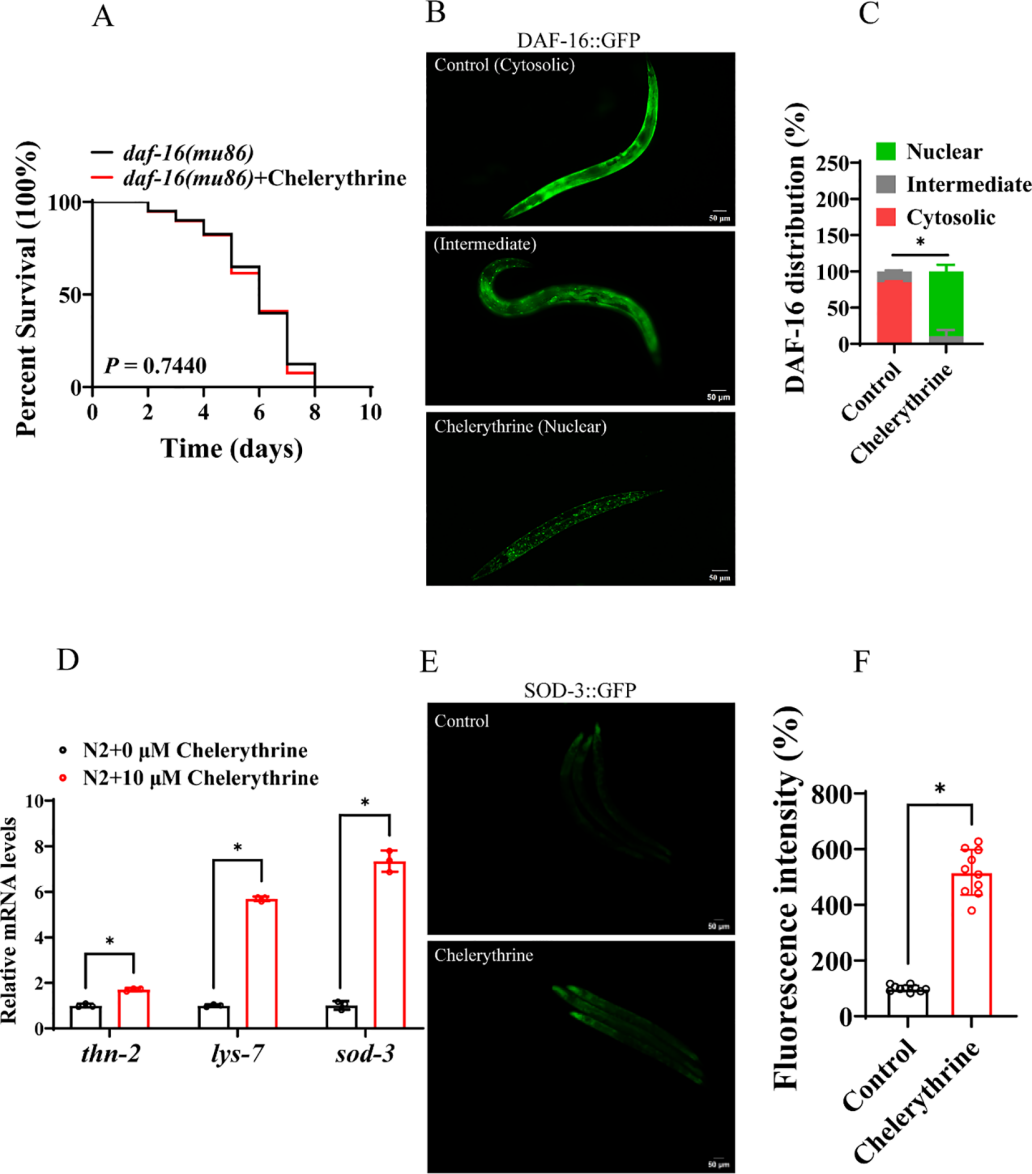
Chelerythrine activates the FoxO transcription factor DAF-16 to promote anti-fungi immunity in *C. elegans*. **(A)** Chelerythrine (10 μM) fails to enhance resistance to *C. albicans* infection in *daf-16(mu86)* mutants (*P* = 0.7440, ns, not significant; n > 40 per group; log-rank test). **(B)** Representative fluorescence images showing the three DAF-16::GFP localization categories: cytosolic (GFP distributed throughout the cytoplasm with little or no nuclear enrichment), intermediate (partial nuclear accumulation with residual cytosolic signal), and nuclear (strong GFP signal concentrated in the nucleus). Scale bar: 50 μm. **(C)** Quantification of DAF-16 subcellular localization, categorized as cytosolic, intermediate, or nuclear (n ≥ 20 per group; *P < 0.05, unpaired t-test). Data are presented as mean ± SEM from three independent experiments. **(D)** Relative mRNA levels of DAF-16 target genes (*sod-3*, *lys-7*, and *thn-2*) in worms treated with or without 10 μM Chelerythrine, determined by qPCR (*P < 0.05, one-way ANOVA). Data are presented as mean ± SEM from three independent experiments. **(E)** Representative fluorescence images showing SOD-3::GFP expression in the SOD-3::GFP transgenic reporter strain (CF1553) treated with or without 10 μM Chelerythrine. Scale bar: 50 μm. **(F)** Quantification of SOD-3::GFP fluorescence intensity (n ≥ 20 per group; *P < 0.05, unpaired t-test).

Previous studies have shown that activated DAF-16 plays a crucial role in innate immunity (22). We found that 10 μM Chelerythrine failed to enhance resistance to *C. albicans* infection in *daf-16(mu86)* mutants compared to WT worms, indicating that DAF-16 is essential for Chelerythrine-mediated immune enhancement (**Figure 4A**). To investigate whether Chelerythrine activates the FoxO transcription factor DAF-16, we monitored its cellular translocation using transgenic worms expressing a functional DAF-16::GFP fusion protein. DAF-16 localization was categorized into three distinct patterns: cytosolic (GFP signal distributed throughout the cytoplasm with little or no nuclear enrichment), intermediate (partial nuclear accumulation accompanied by residual cytosolic signal), and nuclear (strong GFP signal concentrated predominantly in the nucleus) (**Figure 4B**). We observed that 10 μM Chelerythrine significantly induced DAF-16 nuclear localization (**Figures 4B, C**). Next, we tested the expression of DAF-16 target genes, *sod-3*, *lys-7*, and *thn-2 (*23). Quantitative real-time PCR analysis demonstrated that these DAF-16-dependent genes were upregulated in worms treated with 10 μM Chelerythrine (**Figure 4D**). Furthermore, we detected *sod-3* expression using transgenic worms expressing SOD-3::GFP and observed higher GFP fluorescence levels in 10 μM Chelerythrine-treated animals (**Figures 4E, F**). In conclusion, these findings indicate that Chelerythrine activates the Forkhead box O (FoxO) transcription factor DAF-16 in *C. elegans* to enhance pathogens resistance.

### 2.5 Chelerythrine activates the nuclear receptor NHR-49 to enhance anti-fungi immunity in *C. elegans*

**Figure 5:**
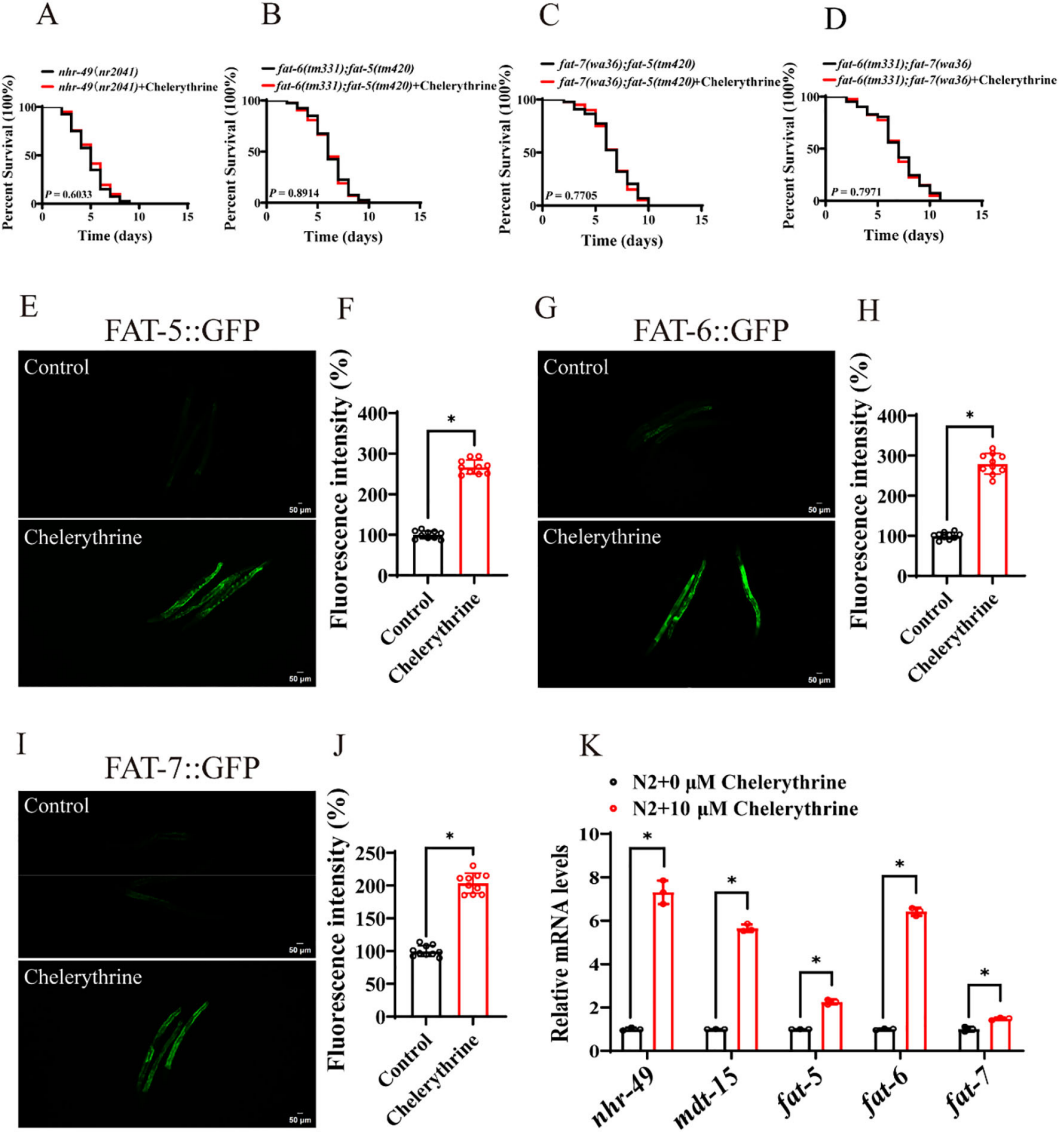
Chelerythrine activates the nuclear receptor NHR-49 to enhance anti-fungi immunity in *C. elegans*. **(A–D)** Chelerythrine (10 μM) fails to enhance resistance to *C. albicans* infection in *nhr-49(nr2041)* mutants **(A)** (*P* = 0.6033, ns, not significant), *fat-5(tm420);fat-6(tm331)* **(B)** (*P* = 0.8914, ns, not significant), *fat-5(tm420);fat-7(wa36)* **(C)** (*P* = 0.7705, ns, not significant), *fat-6(tm331);fat-7(wa36)* double mutants **(D)** (*P* = 0.7971, ns, not significant) after *C. albicans* infection. (n > 40 per group; log-rank test). **(E–J)** Expression of FAT-5::GFP [**(E, F)**, strain BX150], FAT-6::GFP [**(G, H)**, strain BX115], FAT-7::GFP [**(I, J)**, strain BX113] was up-regulated in the respective transgenic reporter strains following exposure to Chelerythrine (10 μM). Scale bars: 50 μm. Right panels show quantification of fluorescence intensity. Data are presented as mean ± SEM of three independent experiments. (n ≥ 20 per group; *P < 0.05, unpaired two-tailed Student’s t-test). **(K)** qPCR analysis of fatty acid metabolism-related genes (*nhr-49*, *mdt-15*, *fat-5*, *fat-6*, *fat-7*) in worms treated with or without 10 μM Chelerythrine. Data are presented as mean ± SEM of three independent biological replicates. (*P < 0.05, one-way ANOVA).

Our transcriptomic analysis revealed significant enrichment of genes associated with fatty acid metabolism pathways in Chelerythrine-treated worms compared with controls (**Figure 3**). Within the *C. elegans* genome, NHR-49 is the principal nuclear hormone receptor orthologous to mammalian PPARα and is established as the master transcriptional regulator of fatty acid metabolism. Based on this enrichment and the central role of NHR-49 in coordinating lipid homeostasis, we selected NHR-49 for further functional validation. To investigate whether Chelerythrine activates the nuclear receptor NHR-49, we examined the survival of *nhr-49(nr2041)* mutants following *C. albicans* infection. We found that 10 μM Chelerythrine failed to enhance resistance to *C. albicans* infection in *nhr-49(nr2041)* mutants compared to WT worms (**Figure 5A**), suggesting that NHR-49 is required for Chelerythrine-mediated immune enhancement. The fat-*5*, *fat-6*, and *fat-7* genes encode three Δ9 fatty acid desaturases that are functionally redundant for monounsaturated fatty acid synthesis. Single mutants of these genes display only subtle changes in fatty acid composition and no visible phenotypes due to compensatory upregulation of the remaining desaturases (24), whereas the *fat-5; fat-6; fat-7* triple mutant is lethal (25). Therefore, to interrogate the functional requirement of the NHR-49/fatty acid metabolism pathway in Chelerythrine-mediated immunity, we employed the viable fat-6; fat-7 double mutant—a strategy that is standard practice in the field. We further tested the core components of fatty acid metabolism, the Δ9 Desaturases of *fat-5(tm420), fat-6(tm331) and fat-7(wa36)*, We also found that 10 μM Chelerythrine could not confer resistance to *C. albicans* infection in double mutants of *fat-5(tm420); fat-6(tm331), fat-5(tm420); fat-7(wa36), fat-6(tm331); fat-7(wa36)*, compared to WT worms (**Figures 5B–D**). Taken together, these results indicate that NHR-49 and its downstream desaturases play a crucial role in Chelerythrine-mediated innate immunity against fungal infection. Next, we examined the expression of fatty acid metabolism-related genes using transgenic worms expressing FAT-5::GFP, FAT-6::GFP, FAT-7::GFP. We observed significantly higher GFP fluorescence levels in worms treated with 10 μM Chelerythrine compared to controls (**Figures 5E–J**). Additionally, quantitative real-time PCR analysis demonstrated that NHR-49-dependent genes, including *nhr-49, mdt-15, fat-5*, *fat-6* and *fat-7*, were upregulated in 10 μM Chelerythrine-treated animals (**Figure 5K**). In conclusion, these findings demonstrate that Chelerythrine engages the nuclear receptor NHR-49 and its downstream fatty acid metabolism pathway in *C. elegans*, establishing their genetic requirement for enhanced pathogen resistance. Whether Chelerythrine directly alters lipid metabolism through modulation of these desaturase activities remains to be investigated by future metabolomic profiling.

### 2.6 Chelerythrine enhances anti-fungi immunity in *C. elegans* independent on canonical immune signaling pathways

**Figure 6:**
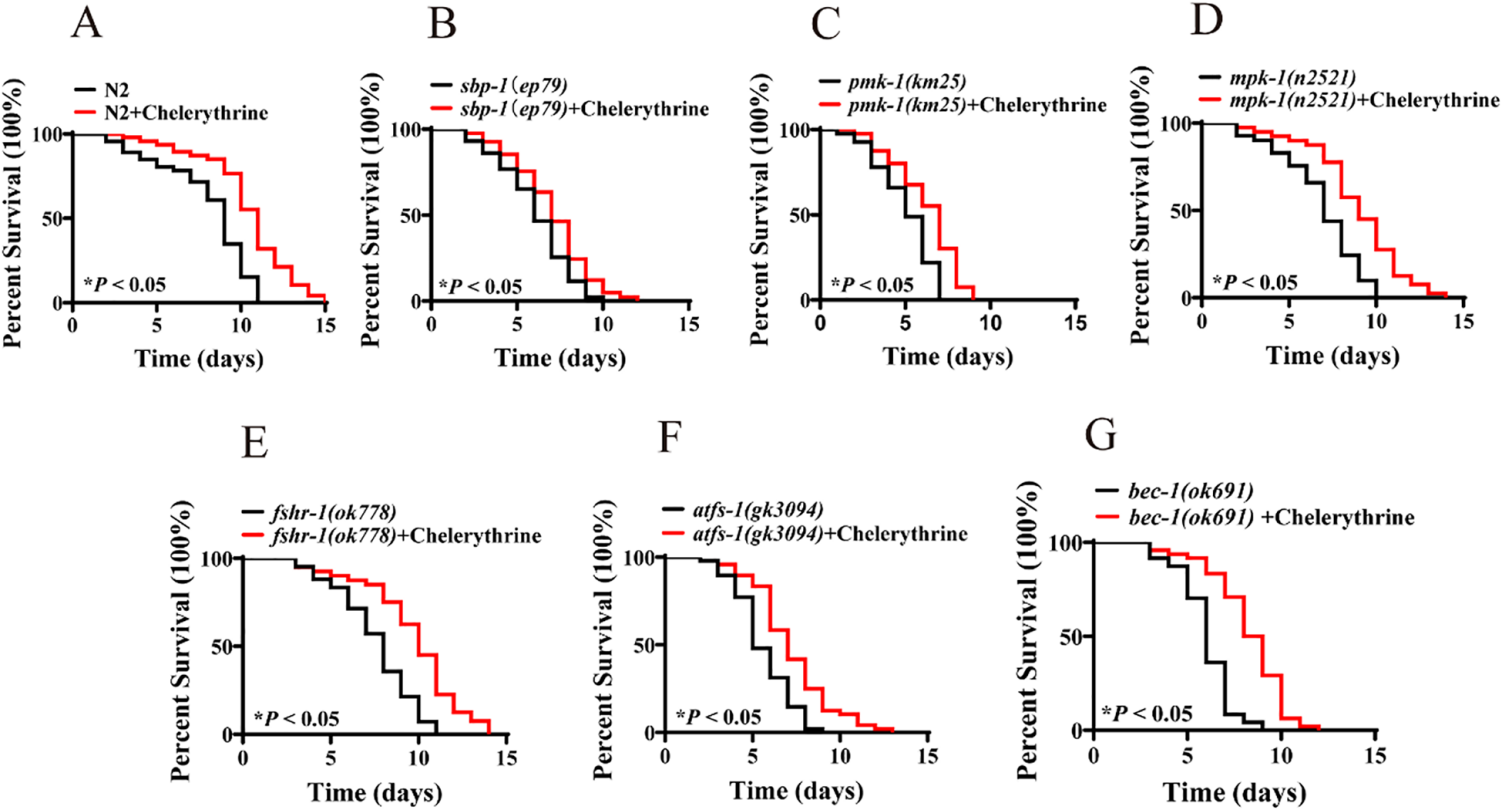
Chelerythrine enhances anti-fungi immunity in *C. elegans* independent on canonical immune signaling pathways. **(A–G)** Chelerythrine enhances resistance to *C. albicans* infection independently of several canonical immune signaling pathways. Following treatment with 10 μM Chelerythrine, the survival rate of N2 wild-type worms **(A)**, *sbp-1(ep79)* **(B)**, *pmk-1(km25)* **(C)**, *mpk-1(n2521)* **(D)**, *fshr-1(ok778)* **(E)**, *atfs-1(gk3094)* **(F)** and *bec-1(ok691)* **(G)** mutants after *C. albicans* infection. (n > 40 per group; *P < 0.05; log-rank test).

To determine the molecular mechanisms by which Chelerythrine confers protection against pathogen infection, we screened several signaling pathways involved in innate immunity in *C. elegans*, including SBP-1/SREBP (26), P38 MAPK/PMK-1 (27), ERK MAPK/MPK-1 (28), FSHR-1 (29), ATFS-1 (30, 31), BEC-1 (32). Treatment with 10 μM Chelerythrine significantly increased the survival rate of wild-type N2 worms following *C. albicans* infection (**Figure 6A**). Notably, this protective effect was retained in mutants of the aforementioned pathways, including *sbp-1(ep79)*, *pmk-1(km25)*, *mpk-1(n2521)*, *fshr-1(ok778), atfs-1(gk3094) and bec-1(ok691)* (**Figures 6B–G**). Taken together, these results demonstrate that Chelerythrine promotes anti-fungi immunity in *C. elegans* independent on canonical immune signaling pathways.

## 3 Discussion

*Candida albicans*, as an opportunistic fungal pathogen, can cause a spectrum of diseases ranging from superficial mucosal infections to life-threatening systemic conditions in immunocompromised hosts (33). It is important to note that *C. albicans* is capable of causing monomicrobial diseases in humans, such as oropharyngeal candidiasis (thrush), in which it serves as the sole etiological agent. The widespread emergence of resistance to azole and other antifungal agents has posed escalating challenges to clinical management (33). In recent years, strategies aimed at enhancing anti-infective capacity through the modulation of host innate immunity have garnered significant attention. Within this context, the discovery of natural products with immunomodulatory properties from traditional medicinal herbs has emerged as a pivotal direction in novel drug development. Chelerythrine, a natural benzophenanthridine alkaloid, has been demonstrated to possess diverse pharmacological activities, including antitumor, anti-inflammatory (13), and antifungal effects (9). However, whether Chelerythrine enhances resistance against *C. albicans* infection through modulation of host immunity, and the underlying molecular mechanisms, have not been previously reported. In this study, utilizing *C. elegans* as a model organism, we reveal for the first time the role and molecular mechanism of Chelerythrine in enhancing host innate immunity against *C. albicans*. Our findings demonstrate that Chelerythrine significantly extends the lifespan of *C. elegans* following *C. albicans* infection and inhibits fungal proliferation. Notably, whereas Chelerythrine exhibits clear antifungal activity *in vitro* (**Figure 1C**), the intestinal fungal burden in the worms remains unchanged (**Figures 1D, E**). We attribute this apparent discrepancy to the following considerations. First, a substantial drug concentration gradient exists between the liquid culture environment and the intestinal lumen. In liquid broth, 10 μM Chelerythrine directly and uniformly contacts the *C. albicans* cell membrane, thereby exerting a sustained fungistatic effect. *In vivo*, however, the intestinal epithelium of *C. elegans* constitutes a natural barrier. As a small-molecule alkaloid, Chelerythrine may be metabolized by intestinal cells during absorption or become bound to food debris and proteins within the gut lumen, resulting in a free luminal drug concentration well below the effective fungistatic threshold—insufficient to inhibit fungal proliferation. Second, a distinction must be drawn between fungistatic activity and host disease tolerance. The *in vitro* data in **Figure 1C** demonstrate that Chelerythrine acts by suppressing fungal cell division and population expansion (fungistasis) rather than by direct killing (fungicidal activity). Moreover, this fungistatic effect was largely overcome after approximately 8 hours, with the growth curve of the Chelerythrine-treated group eventually approaching that of the control group. This transient nature of the inhibition is relevant to our infection assay design: *C. albicans* was spread onto NGM plates and incubated overnight (approximately 24 hours) to form a uniform lawn before worms were introduced. Consequently, any drug-induced suppression of fungal growth on the plate surface would have largely subsided during the 24-hour pre-incubation period, minimizing the potential confounding effect of pre-ingestion fungal inhibition. The innate immune strategies employed by *C. elegans* against pathogens primarily include pathogen clearance, enhanced tolerance, and avoidance behavior (34, 35). The unaltered intestinal CFU counts observed in this study directly exclude the possibility that Chelerythrine enhances intestinal clearance capacity. Instead, the marked improvement in worm survival (**Figure 1B**) in the absence of reduced internal pathogen load strongly points to enhanced host immune tolerance—that is, the worms withstand the pathological damage inflicted by a high fungal burden through augmentation of their intrinsic innate immune defenses. This phenotypic signature is consistent with a model in which the protective effect of Chelerythrine is mediated predominantly through host-directed mechanisms rather than through attenuation of pathogen virulence. Importantly, this tolerance phenotype is mechanistically distinct from the survival advantage conferred by classic longevity mutations. In canonical longevity mutants such as *daf-2* and *age-1*, constitutive DAF-16 activation pre-upregulates basal immunity even in the absence of infection. Upon pathogen exposure, these mutants can rapidly clear invading microbes, resulting in reduced pathogen burden coupled with increased survival. This classic longevity-to-resistance causal chain—longevity mutation → constitutive DAF-16 activation → elevated basal immunity → enhanced pathogen clearance → reduced pathogen burden → increased survival—predicts that if Chelerythrine acted merely by extending lifespan through DAF-16, we would observe both increased survival and reduced fungal burden. The absence of CFU reduction in our study therefore argues against a simple longevity extension model and instead supports a model of infection-specific immune tolerance enhancement. Finally, although pre-ingestion contact between the drug and the pathogen on the NGM agar surface might theoretically induce a minor degree of pre-ingestion inhibition, the restricted diffusion of the drug in solid medium, the high-density inoculum effect of the fungal lawn, and the transient nature of Chelerythrine’s fungistatic activity (with growth resuming after 8 hours) render the contribution of this surface effect to the multi-day host protection phenotype negligible. We acknowledge, however, that the direct effects of Chelerythrine on *C. albicans* pathogenic traits such as hyphal formation, adhesion capacity, and virulence factor expression were not assessed in this study. Systematically characterizing these effects would provide a more comprehensive understanding of the host-pathogen-drug tripartite interaction and would represent a valuable direction for future investigation. Taken together, these findings indicate that the protective effect of Chelerythrine in *C. elegans* is governed predominantly by the enhancement of host innate immune responses rather than by direct environmental antifungal action.

Additionally, it is worth noting that in the context of *C. albicans* infection, 100 μM fat conferred significantly less protection than 10 μM (**Figure 1B**). We wish to clarify that this U-shaped dose-response profile was observed specifically under infection conditions. We acknowledge that the present study did not include a comprehensive evaluation of the basal toxicological profile of Chelerythrine. We do not assert that 100 μM is inherently toxic to uninfected worms; rather, we interpret this finding to indicate that this concentration represents the upper limit of the therapeutic window under pathogenic stress. A more detailed characterization of the dose-toxicity relationship across a broader concentration range awaits further investigation in future work.

Furthermore, we examined whether the protective effect of Chelerythrine extends beyond *C. albicans* to bacterial pathogens. As shown in **Figures 2A–D**, treatment with 10 μM Chelerythrine significantly prolonged the lifespan of *C. elegans* infected with *Listeria monocytogenes*, *Enterococcus faecalis*, *Pseudomonas aeruginosa*, and *Salmonella enterica*. Notably, the same concentration of Chelerythrine did not suppress the *in vitro* proliferation of these bacterial strains (**Figures 2E–H**). Collectively, these data indicate that Chelerythrine confers a broad-spectrum enhancement of host resistance. Future studies will be required to validate the specific immune mechanisms involved during bacterial infections.

More importantly, Chelerythrine promotes innate immunity in *C. elegans* through the conserved FoxO signaling pathway and fatty acid metabolism pathway. Deletion mutations of *daf-16* and *nhr-49* completely abrogate the lifespan-extending effect of Chelerythrine in infected nematodes, demonstrating their indispensable roles in the immunoenhancing activity of this compound.

DAF-16, a core transcription factor of the FoxO pathway (36) and a key downstream target of the insulin signaling pathway, plays a critical role in regulating antioxidant defense, antimicrobial peptide expression, and lifespan extension. Our study shows that Chelerythrine promotes DAF-16 nuclear translocation and upregulates the expression of its downstream target genes *sod-3*, *thn-2*, and *lys-7*. It is important to note that DAF-16 functions as both a key regulator of innate immunity and a well-established modulator of longevity. Nevertheless, several lines of evidence from the present study indicate that the protective effect of Chelerythrine under infection conditions primarily reflects enhanced immune tolerance rather than a simple extension of basal lifespan. First, RT-qPCR and fluorescent reporter gene assays confirmed that Chelerythrine specifically upregulates antifungal immune effectors, such as *sod-3*, *thn-2*, and *lys-7*, in an infection-dependent manner. Second, the cooperative involvement of the NHR-49/fatty acid metabolism pathway further supports a host metabolic reprogramming response rather than a generalized longevity reaction. Third, as discussed above, the unaltered intestinal fungal burden despite significantly improved survival is a defining feature of immune tolerance, which contrasts with the reduced pathogen burden typically observed in longevity mutants. Although we acknowledge that a lifespan assay in uninfected worms would provide a valuable baseline control—and we recognize the absence of such an experiment as a limitation of the current study—the available phenotypic data strongly support the conclusion that Chelerythrine enhances host innate immunity specifically in the context of *C. albicans* infection. Our KEGG pathway enrichment analysis revealed that the Chelerythrine-treated group exhibited significant enrichment of genes associated with the longevity regulating pathway, an intriguing observation that merits further investigation. Future studies will aim to delineate the impact of Chelerythrine on *C. elegans* healthspan in the absence of pathogenic stress.

Notably, the protective effect of Chelerythrine in nematodes is also dependent on the nuclear receptor NHR-49 (17) and its downstream desaturase genes *fat-5*, *fat-6*, and *fat-7*. As the functional ortholog of mammalian PPARα, NHR-49 primarily maintains lipid homeostasis by regulating fatty acid β-oxidation and desaturation, with recent studies progressively revealing its crucial role in immune regulation (37). Fatty acid metabolism not only provides energy and membrane structural foundations for immune responses, but its metabolites, such as unsaturated fatty acids, can also serve as signaling molecules directly modulating immune-related gene expression. Our finding that NHR-49 and its downstream desaturase genes mediate the immunoenhancing effects of Chelerythrine suggests that this compound may also enhance immune defense through modulation of lipid metabolism. However, we acknowledge that our functional validation of core genes was performed using only single-allele loss-of-function mutants without rescue experiments or tissue-specific RNAi verification, which represents a limitation of the current study. Although the consistency of phenotypes across multiple independent mutant alleles (*nhr-49* and three distinct *fat* double mutant combinations) substantially reduces the likelihood of background mutation interference, future rescue experiments would further consolidate the genotype-phenotype causality. Although we have demonstrated that Chelerythrine activates NHR-49 and its downstream target genes, we fully acknowledge that the precise molecular mechanism by which Chelerythrine leads to NHR-49 activation—whether through direct ligand binding, modulation of upstream signaling cascades, or indirect metabolic sensing—remains an open and important question. Identifying the direct molecular target of Chelerythrine and elucidating the detailed mechanism of NHR-49 activation represent key directions for future investigation. Furthermore, while our data establish that the *fat-5/6/7* desaturases play a critical role in mediating Chelerythrine’s antifungal immunity, and that their expression is transcriptionally upregulated by Chelerythrine treatment, whether Chelerythrine directly alters the lipid metabolic profile of *C. elegans* through modulation of these desaturase activities remains to be determined by future metabolomic profiling.

Importantly, our study suggests a potential functional interaction between DAF-16 and NHR-49 in response to *C. albicans* infection (17). Evidence indicates that DAF-16 can regulate the expression of certain lipid metabolism-related genes, while the lipid homeostasis maintained by NHR-49 provides the necessary membrane environment and signaling molecules for DAF-16-mediated stress defense (38). The concurrent activation of both pathways by Chelerythrine suggests that it may function as an upstream signal that coordinately regulates the immune-metabolic network through as-yet-undefined mechanisms. This mechanism of action differs from previously reported studies in which various natural products and pharmaceuticals extend nematode lifespan through classical immune signaling pathways. For instance, metformin enhances innate immunity in both *C. elegans* and mice via p38 MAPK signaling (29); luteolin promotes pathogen resistance in *C. elegans* in a DAF-2/DAF-16 insulin-like signaling pathway-dependent manner; sanguinarine promotes healthspan and enhances innate immunity through PMK-1/SKN-1-dependent mechanisms (39); brevilin A promotes innate immunity in *C. elegans* in a p38 MAPK-dependent manner (40); and aspartame enhances innate immunity and extends lifespan in *C. elegans* via the autophagy pathway (32). Collectively, Chelerythrine possesses a distinct mechanism of action, and future investigations are warranted to explore its direct molecular targets and the specific interactive mechanisms between DAF-16 and NHR-49.

**Figure 7:**
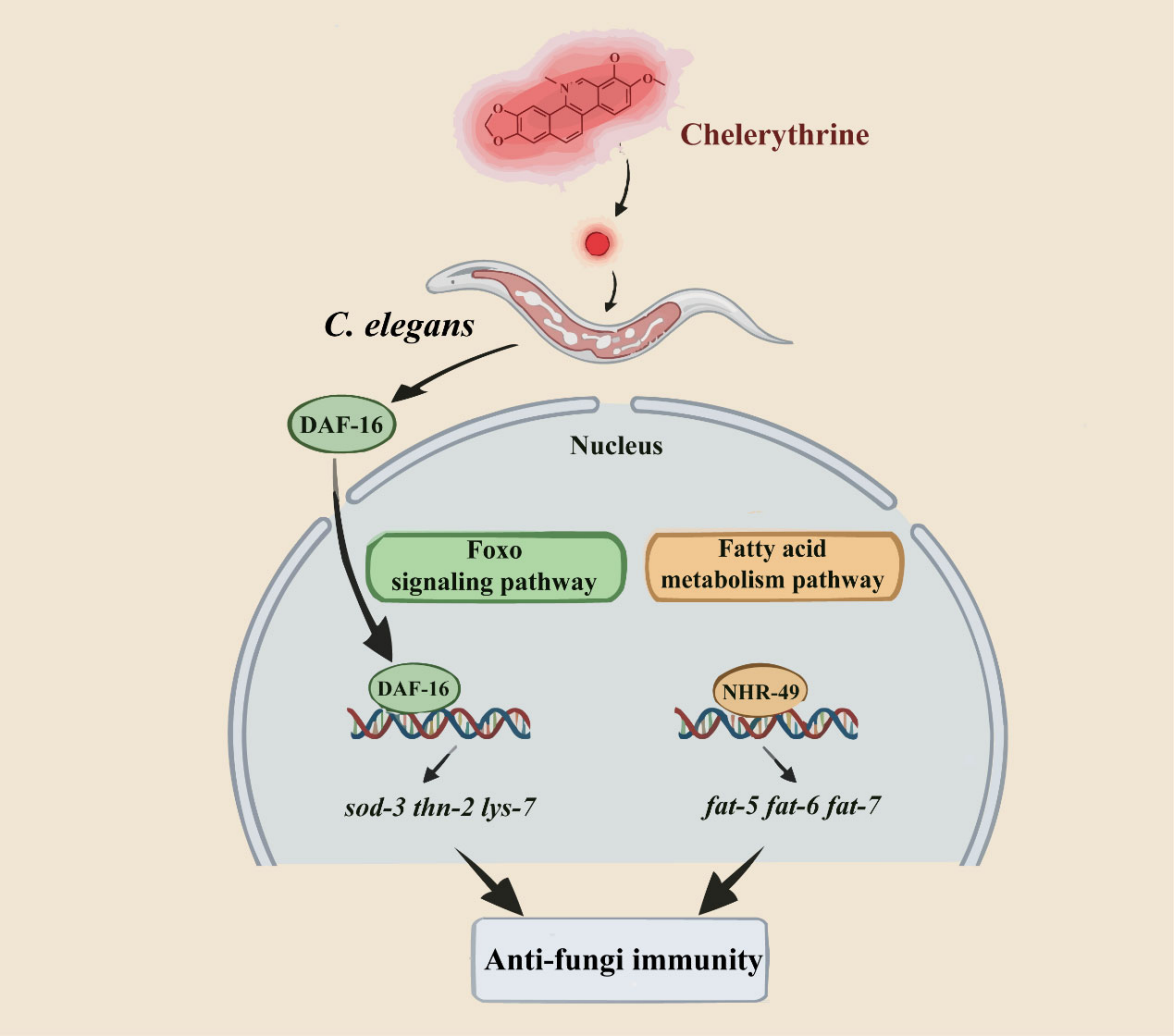
Schematic diagram illustrates the mechanism by which Chelerythrine enhances anti-fungi immunity in *C. elegans* via the FoxO signaling and fatty acid metabolism pathways.

In summary, this study demonstrates that Chelerythrine enhances the innate immune response of *C. elegans* against *C. albicans* by activating the DAF-16-mediated FoxO pathway and the NHR-49-mediated fatty acid metabolism pathway (**Figure 7**). Given the evolutionary conservation of the FoxO pathway and fatty acid metabolism pathways in mammals, Chelerythrine-a natural product that enhances host defense through simultaneous modulation of immune and metabolic networks-may offer greater efficacy and a lower risk of drug resistance compared to single-target agents, thereby providing novel insights for the development of therapeutic strategies against fungal infections. Subsequent studies should further validate its immunoenhancing effects and safety profile in mammalian models and explore its potential as an adjunctive therapeutic agent in clinical applications.

## 4 Materials and methods

### 4.1 Chemicals

Chelerythrine (purity ≥ 98%, as determined by HPLC) was obtained from Sigma Chemical Co. (St. Louis, MO, USA), and was dissolved in dimethyl sulfoxide (DMSO) as a stock solution at a 10 mM concentration and was stored in aliquots at -20 °C.

### 4.2 Worm strains and cultivation

Worms were maintained and propagated under standard conditions as previously described (41–43). The following nematode strains were obtained from the Caenorhabditis Genetics Center (CGC), which was funded by NIH Office of Research Infrastructure Programs (P40 OD010440): N2 Bristol wild-type, CF1038 *daf-16 (mu86)*, STE68 *nhr-49 (nr2041)*, CE541 *sbp-1(ep79)*, BX110 *fat-5(tm420); fat-6(tm331)*, BX160 *fat-5(tm420); fat-7(wa36)*, BX156 *fat-6(tm331); fat-7(wa36)*, KU25 *pmk-1 (km25)*, SD184 *mpk-1(n2521)*, RB911 *fshr-1(ok778)*, VC3201 *atfs-1(gk3094)*, VC517 *bec-1(ok691)*, TJ356 (DAF-16::GFP), CF1553 (SOD-3::GFP), BX150 (FAT-5::GFP), BX115 (FAT-6::GFP), and BX113 (FAT-7::GFP). *C. elegans* mutants were backcrossed three times to the wild-type(N2) strain and used in the laboratory.

### 4.3 Infection assay

*E. coli* OP50, *L. monocytogenes* 10403S, *S. enterica* SL1344, *E. faecalis* ATCC 29212, and *P. aeruginosa* PA14 were grown overnight in LB broth at 37 °C. *C. albicans* SC5314 was grown overnight in Sabouraud Dextrose Broth (SDA) at 30 °C. For plate preparation, 500 μL of each overnight pathogen culture was spread onto 90 mm nematode growth medium (NGM) agar plates. The plates were then incubated overnight at the respective growth temperature (37 °C for bacterial pathogens, 30 °C for *C. albicans*) to establish a uniform microbial lawn. All cultures were then spread onto nematode growth medium (NGM) plates. All infection assays were performed on NGM agar plates or NGM plates supplemented with or without Chelerythrine (0, 1, 10, 100 μM). Chelerythrine was dissolved in dimethyl sulfoxide (DMSO) to prepare a 10 mM stock solution. For dose-screening experiments, NGM agar plates were prepared with a total volume of 50 mL per group, with the following additions to achieve the indicated final concentrations while maintaining a constant final DMSO volume of 500 μL (1%) across all groups: 0 μM Chelerythrine, 500 μL DMSO; 1 μM Chelerythrine, 5 μL stock + 495 μL DMSO; 10 μM Chelerythrine, 50 μL stock + 450 μL DMSO; 100 μM Chelerythrine, 500 μL stock (44). For all subsequent mechanistic experiments, in which only the optimal concentration of 10 μM Chelerythrine was evaluated, the DMSO volume was reduced to 50 μL per group (0.1% final concentration) for both the control (50 μL DMSO) and the 10 μM Chelerythrine group (50 μL stock) (44). This solvent compensation strategy ensures that control and treated groups are exposed to identical final DMSO concentrations within each experimental phase, thereby excluding solvent-related confounding effects. Synchronized populations of worms were cultivated on *E. coli* OP50 at 20 °C until the young adult stage. 30–60 worms were transferred to NGM agar plates containing *L. monocytogenes* 10403S, *S. enterica* SL1344, *E. faecalis* ATCC 29212, *P. aeruginosa* PA14, and *C. albicans* SC5314 at 25 °C, respectively. The number of living worms was counted at 24-hour intervals. Immobile adult worms unresponsive to touch were scored as dead (45–47). Three plates were tested per assay, and all experiments were performed three times independently.

### 4.4 Fungal proliferation assay

*C. albicans* SC5314 was grown overnight in Sabouraud Dextrose Broth at 30 °C with shaking at 200 rpm. The overnight culture typically reached an OD_600_ of approximately 1.0–1.5. The culture was then diluted 1,000-fold in fresh SDA (pH 7.0) (48, 49) to a starting OD_600_ of ~0.001–0.0015. This diluted culture was aliquoted into microtiter plates, and 10 μM Chelerythrine or an equivalent volume of DMSO (vehicle control) was added. Plates were incubated at 30 °C with continuous shaking at 200 rpm. Absorbance at 600 nm was measured every 2 hours over a 24-hour period using a microplate reader. All OD_600_ values were corrected by subtracting the absorbance of a blank well containing SDA medium only. Data were collected from three independent replicates for each condition.

### 4.5 Bacterial proliferation assay

Bacterial strains were grown overnight in LB broth at 37 °C with shaking at 180 rpm. The typical OD_600_ of overnight cultures prior to dilution was as follows: approximately 1.0–1.5 for *L. monocytogenes*, *P. aeruginosa*, and *S. enterica*; approximately 0.5 for *E. faecalis*. Cultures were then diluted 1,000-fold in fresh LB (pH 7.0) (50, 51) to an estimated starting OD_600_ of ~0.001–0.0015 for most strains, and ~0.0005 for *E. faecalis*. The diluted cultures were aliquoted into microtiter plates, and 10 μM Chelerythrine or an equivalent volume of DMSO (vehicle control) was added. Plates were incubated at 37 °C with continuous shaking at 180 rpm. Absorbance at 600 nm was measured every 2 hours over a 24-hour period. All OD_600_ values were corrected by subtracting the absorbance of a blank well containing LB medium only. Data were collected from three independent replicates for each condition.

### 4.6 Quantification of intestinal fungal loads

Synchronized populations of worms were cultivated on *E. coli* OP50 at 20 °C until the young adult stage. *C. albicans* were grown in SDA liquid medium at 30 °C overnight. For plate preparation, 500 μL of the overnight *C. albicans* culture was spread onto 90 mm NGM agar plates, and the plates were incubated overnight at 30 °C to establish a uniform fungal lawn. Worms were then transferred to NGM agar plates (supplemented with or without 10 μM Chelerythrine) containing *C. albicans* for 48 h at 25 °C (52, 53). To eliminate the *C. albicans* from the surface of worms, worms were transferred to NGM agar plate seeded with *E. coli* OP50 for 20 min, and this step was repeated three times (54). Twenty worms were transferred into 200 μl PBS plus 0.1% Triton and ground (54). The lysates were spread onto SDA agar plates and incubated at 30 °C. After two days of incubation at 30 °C, colonies of *C. albicans* were counted. Three plates were tested per assay and all experiments were performed three times independently.

### 4.7 RNA-seq data analysis

For RNA-sequencing, samples were collected under *C. albicans* infection conditions. Specifically, wild-type N2 worms were infected with C. albicans and simultaneously treated with 10 μM Chelerythrine or vehicle control for 48 hours prior to RNA extraction, thereby capturing transcriptomic changes that occur during active infection. Briefly, synchronized L4-stage wild-type N2 worms were transferred to NGM plates containing a *C. albicans* lawn supplemented with 10 μM Chelerythrine or vehicle control and incubated at 25 °C for 48 hours. Approximately 500 μL of packed worms were collected per replicate for each sample, and three independent biological replicates were prepared for each treatment group. Total RNA was extracted using Trizol reagent (ThermoFisher, 15596018) following the manufacturer’s instructions. The average insert size for the final cDNA libraries were 300 ± 50 bp. Finally, 2 × 150 bp paired-end sequencing (PE150) was performed on an Illumina NovaSeq 6000 platform (LC-BioTechnology CO., Ltd., Hangzhou, China) according to the vendor’s recommended protocol. This Illumina paired-end RNA-seq method generated millions of 2 × 150 bp paired-end reads. All transcriptomes from all samples were merged to reconstruct a comprehensive transcriptome using gffcompare software. After the final transcriptome was generated, StringTie and ballgown were used to estimate the expression levels of all transcripts and to calculate mRNA expression abundance based on FPKM (fragments per kilobase of transcript per million mapped reads) values. Differential gene expression analysis was conducted using DESeq2 for group comparisons and edgeR for individual sample comparisons. Genes with false discovery rate (FDR) < 0.05 and | log_2_FoldChange | ≥ 0.5 were considered differentially expressed. Differentially expressed genes were then subjected to Gene Ontology (GO) functional enrichment analysis and Kyoto Encyclopedia of Genes and Genomes (KEGG) pathway enrichment analysis. The raw sequencing data have been submitted to the NCBI Gene Expression Omnibus (GEO) under the accession number PRJNA1401773.

### 4.8 Fluorescence microscopy

Synchronized L1 worms of the DAF-16::GFP, SOD-3::GFP, FAT-5::GFP, FAT-6::GFP and FAT-7::GFP strains were cultivated on NGM plates seeded with *E. coli* OP50 at 20 °C until they reached the L4 stage. L4-stage worms were then transferred to NGM agar plates containing a *C. albicans* lawn (prepared as described in Section 4.3) supplemented with or without 10 μM Chelerythrine and incubated at 25 °C for 12 hours. Images were obtained using a Zeiss Axioskop 2 plus fluorescence microscope (Carl Zeiss, Jena, Germany) equipped with a digital camera. Fluorescence intensity was quantified using the ImageJ (55) software (NIH). Three plates with approximately 30 animals per plate were examined per assay, and all experiments were performed independently three times.

### 4.9 Quantitative real-time PCR

Nematodes were synchronized and treated for 2 days with or without 10 μM Chelerythrine starting at the L4 larval stage. Total RNA was extracted from worms using TRIzol Reagent (Invitrogen) as previously described (55, 56). Random-primed cDNAs were generated by reverse transcription of the total RNA samples with SuperScript II (Invitrogen). qPCR was performed using SYBR Premix Ex Tag (Takara, Dalian, China) on an Applied Biosystems Prism 7000 Sequence Detection System (Applied Biosystems, Foster City, CA, USA). Using *pmp-3* for an internal control as previously described (57). The following primers were used in this study:

*pmp-3* primers:

*pmp-3*-F: TGGATTGTCATTGGCGTCG.

*pmp-3*-R: GTTGTCGCAGAGTGGTGTTT.

*sod-3* primers:

*sod-3*-F: TCCAAGCACACTCTCCCAGAT.

*sod-3*-R: TCTCCACCATCCTTAGCCAAG.

*thn-2* primers:

*thn-2*-F: GCTCGCACCATCACTATCTAC.

*thn-2*-R: CACATCCAGTTCTTGCCCAA.

*lys-7* primers:

*lys-7*-F: ATGACTCCACAGCCCGTTT.

*lys-7*-R: GGCGAAGTGACCTGAATCCA.

*nhr-49* primers:

*nhr-49*-F: GTCGTTATTGTCGCTTTCAA

*nhr-49*-R: TCCGACACCGTTGCTGTTTC

*mdt-15* primers:

*mdt-15*-F: CGTAGCAACAACACAGGCATCAAC

*mdt-15*-R: AACAGCAGCAGTGGCAGAAGC

*fat-5* primers:

*fat-5*-F: GGGCTACAGTTGGATGGGTATT

*fat-5*-R: CGGGTCAGCATCAGTATCCG

*fat-6* primers:

*fat-6*-F: AAGATTGAGAAGGACGGCGG

*fat-6*-R: TCACGGTTTGCCATTTTGCC

*fat-7* primers:

*fat-7*-F: AAGGAGCATGGAGGCAAACT

*fat-7*-R: TTCTCAACGGCGGAAACAGA

### 4.10 Statistics

Data were presented as mean ± SEM. Statistical analyses for all data except for survival assays was carried out using Student’s t-test (unpaired, two-tailed) or ANOVA after testing for equal distribution of the data and equal variances within the data set. Survival data were analyzed by using the log-rank (Mantel-Cox) test. All statistical analyses and data plotting were performed using GraphPad Prism (GraphPad Software, San Diego, CA, USA). *P* < 0.05 was considered significant. .

## Data Availability

The datasets presented in this study can be found in online repositories. The names of the repository/repositories and accession number(s) can be found below: https://www.ncbi.nlm.nih.gov/geo/, PRJNA1401773.

## References

[1] Salvatori O Puri S Tati S Edgerton M . Innate immunity and saliva in candida albicans–mediated oral diseases. J Dent Res. (2016) 95:365–71. doi: 10.1177/0022034515625222 PMC4802782 26747422

[2] Pappas PG Lionakis MS Arendrup MC Ostrosky-Zeichner L Kullberg BJ . Invasive candidiasis. Nat Rev Dis Primers. (2018) 4:18026. doi: 10.1016/j.idc.2006.07.004 29749387

[3] Tsai MH Hsu JF Chu SM Chang PJ Lai MY Wu IH . Clinical and microbiological characteristics, and impact of therapeutic strategies on the outcomes of children with candidemia. Sci Rep. (2017) 7:1083. doi: 10.1038/s41598-017-01123-6 28439070 PMC5430948

[4] Gow NAR Johnson C Berman J Coste AT Cuomo CA Perlin DS . The importance of antimicrobial resistance in medical mycology. Nat Commun. (2022) 13:5352. doi: 10.1038/s41467-022-32249-5 36097014 PMC9466305

[5] Fisher MC Alastruey-Izquierdo A Berman J Bicanic T Bignell EM Bowyer P . Tackling the emerging threat of antifungal resistance to human health. Nat Rev Microbiol. (2022) 20:557–71. doi: 10.1038/s41579-022-00720-1 35352028 PMC8962932

[6] Katsipoulaki M Stappers MHT Malavia-Jones D Brunke S Hube B Gow NAR . Candida albicans and candida glabrata: global priority pathogens. Microbiol Mol Biol Reviews: MMBR. (2024) 88:e00021. doi: 10.1128/mmbr.00021-23 38832801 PMC11332356

[7] Pukkila-Worley R Ausubel FM Mylonakis E . Candida albicans infection of caenorhabditis elegans induces antifungal immune defenses. PloS Pathog. (2011) 7:e1002074. doi: 10.1371/journal.ppat.1002074 21731485 PMC3121877

[8] Zhou Y Wang Z Zhao H Liu Y Lin Y Zhang J . Chelerythrine inhibits esophageal squamous cell carcinoma progression via PINK1-parkin-mediated mitophagy. J Transl Med. (2025) 23:1116. doi: 10.1186/s12967-025-07025-w 41107946 PMC12532471

[9] Qian W Yang M Li X Sun Z Li Y Wang X . Anti-microbial and anti-biofilm activities of combined chelerythrine-sanguinarine and mode of action against candida albicans and cryptococcus neoformans *in vitro*. Colloids Surfaces B Biointerfaces. (2020) 191:111003. doi: 10.1016/j.colsurfb.2020.111003 32276211

[10] Wei QH Cui DZ Liu XF Chai YY Zhao N Wang JY . *In vitro* antifungal activity and possible mechanisms of action of chelerythrine. Pestic Biochem Physiol. (2020) 164:140–8. doi: 10.1016/j.pestbp.2020.01.007 32284120

[11] Loe MWC Lee RCH Chin WX Min N Teo ZY Ho SX . Chelerythrine chloride inhibits zika virus infection by targeting the viral NS4B protein. Antiviral Res. (2023) 219:105732. doi: 10.1016/j.antiviral.2023.105732 37832876

[12] Zhu M Niu J Jiang J Dong T Chen Y Yang X . Chelerythrine inhibits the progression of glioblastoma by suppressing the TGFB1-ERK1/2/Smad2/3-snail/ZEB1 signaling pathway. Life Sci. (2022) 293:120358. doi: 10.1016/j.lfs.2022.120358 35092731

[13] Cai J Zhang LC Zhao RJ Pu LM Chen KY Nasim AA . Chelerythrine ameliorates rheumatoid arthritis by modulating the AMPK/mTOR/ULK-1 signaling pathway. Phytomed: Int J Phytotherapy Phytopharmacol. (2022) 104:154140. doi: 10.1016/j.phymed.2022.154140 35752081

[14] Brenner S . The genetics of Caenorhabditis elegans. Genetics. (1974) 77:71–94. doi: 10.1093/oxfordjournals.bmb.a071019 4366476 PMC1213120

[15] Madende M Albertyn J Sebolai O Pohl CH . Caenorhabditis elegans as a model animal for investigating fungal pathogenesis. Med Microbiol Immunol. (2020) 209:1–13. doi: 10.1007/s00430-019-00635-4 31555911

[16] Breger J Fuchs BB Aperis G Moy TI Ausubel FM Mylonakis E . Antifungal chemical compounds identified using a C. elegans pathogenicity assay. PloS Pathog. (2007) 3:e18. doi: 10.1371/journal.ppat.0030018 17274686 PMC1790726

[17] Liu F Hong CA Gong S Fan Z Xiao X Xiao Y . Luteolin decreases fat accumulation and extends lifespan in caenorhabditis elegans via DAF-16/FOXO and NHR-49/PPAR-α. J Agric Food Chem. (2025) 73:30749–60. doi: 10.1021/acs.jafc.5c08997 41261365

[18] Zhang SS Gu XM Liu H Zhou YF Cai HL Gu JY . Ginkgolide B promotes fat-lowering and lifespan in caenorhabditis elegans via DAF-2/DAF-16 signaling pathway. J Funct Foods. (2025) 126:106708. doi: 10.1016/j.jff.2025.106708 38826717

[19] Xiao Y Wei L Xiong XF Yang M Sun L . Dioscin integrates regulation of monosaturated fatty acid metabolism to extend the life span through XBP-1/SBP-1 dependent manner. iScience. (2023) 26:106265. doi: 10.1016/j.isci.2023.106265 36936783 PMC10014289

[20] Liu F Zhang H Wang H Zhu X Li S Jiang N . The homeodomain transcription factor CEH-37 regulates PMK-1/p38 MAPK pathway to protect against intestinal infection via the phosphatase VHP-1. Cell Mol Life Sci: CMLS. (2023) 80:312. doi: 10.1007/s00018-023-04970-x 37796333 PMC11072455

[21] Xiao Y Liu F Wu Q Zhu X Yu C Jiang N . Dioscin activates endoplasmic reticulum unfolded protein response for defense against pathogenic bacteria in caenorhabditis elegans via IRE-1/XBP-1 pathway. J Infect Dis. (2024) 229:237–44. doi: 10.1093/infdis/jiad294 37499184

[22] Xiao Y Zhang L Zhu X Qin Y Yu C Jiang N . Luteolin promotes pathogen resistance in caenorhabditis elegans via DAF-2/DAF-16 insulin-like signaling pathway. Int Immunopharmacol. (2023) 115:109679. doi: 10.1016/j.intimp.2023.109679 36640711

[23] Xiao Y Zhang H Li X Han C Liu F . DEAD-box RNA helicase DDX-23 mediates dietary restriction induced health span in caenorhabditis elegans. GeroScience. (2025) 47:153–65. doi: 10.1007/s11357-024-01434-3 39578298 PMC11872819

[24] Anderson SM Cheesman HK Peterson ND Salisbury JE Soukas AA Pukkila-Worley R . The fatty acid oleate is required for innate immune activation and pathogen defense in caenorhabditis elegans. PloS Pathog. (2019) 15:e1007893. doi: 10.1371/journal.ppat.1007893 31206555 PMC6597122

[25] Clark JF Savage-Dunn C . Delta-9 fatty acid desaturase mutants display increased body size. microPub Biol. (2018), 10.17912/SS8E–6587. 10.17912/SS8E-6587 PMC7282526 32550399

[26] Yang F Vought BW Satterlee JS Walker AK Jim Sun ZY Watts JL . An ARC/mediator subunit required for SREBP control of cholesterol and lipid homeostasis. Nature. (2006) 442:700–4. doi: 10.1038/nature04942 16799563

[27] Xiao Y Zhou H Cui Y Zhu X Li S Yu C . Schisandrin a enhances pathogens resistance by targeting a conserved p38 MAPK pathway. Int Immunopharmacol. (2024) 128:111472. doi: 10.1016/j.intimp.2023.111472 38176342

[28] Nicholas HR Hodgkin J . The ERK MAP kinase cascade mediates tail swelling and a protective response to rectal infection in C. elegans. Curr Biol. (2004) 14:1256–61. doi: 10.1016/j.cub.2004.07.022 15268855

[29] Xiao Y Liu F Li S Jiang N Yu C Zhu X . Metformin promotes innate immunity through a conserved PMK-1/p38 MAPK pathway. Virulence. (2020) 11:39–48. doi: 10.1080/21505594.2019.1706305 31851866 PMC6961722

[30] Xiong J Li X Mao F Wang N Liu F Xiao Y . Ursolic acid activates mitochondrial unfolded protein response to enhance innate immunity via transcription factor ATFS-1/ATF5. J Agric Food Chem. (2025) 73:29629–37. doi: 10.1021/acs.jafc.5c09928 41213894

[31] Zhang Y Li X Zhao R Hu W Xiao X Xiao Y . Mitochondrial UPR is required for resveratrol mediated anti-bacterial immunity. Food Funct. (2025) 16:8604–15. doi: 10.1039/d5fo03539b 41105143

[32] Han C Luo J Ding L Xiao X Liu F Xiao Y . Aspartame enhances innate immunity and extends lifespan in caenorhabditis elegans via autophagy pathway. Food Res Int. (2026) 227:118231. doi: 10.1016/j.foodres.2025.118231 41652761

[33] Wang Y . Looking into candida albicans infection, host response, and antifungal strategies. Virulence. (2015) 6:307–8. doi: 10.1080/21505594.2014.1000752 PMC4601349 25590793

[34] Zheng Z Zhang X Liu J He P Zhang S Zhang Y . GABAergic synapses suppress intestinal innate immunity via insulin signaling in caenorhabditis elegans. PNAS. (2021) 118:e2021063118. doi: 10.1007/978-981-99-8929-4_3 33972423 PMC8157918

[35] Irazoqui JE Urbach JM Ausubel FM . Evolution of host innate defence: insights from caenorhabditis elegans and primitive invertebrates. Nat Rev Immunol. (2010) 10:47–58. doi: 10.1038/nri2689 20029447 PMC2965059

[36] Zhang H Xiong J Wang Q Song Q Meng L Zhang H . Chrysophanol delays aging via insulin/IGF-1 signaling pathway. Free Radical Biol Med. (2025) 232:269–78. doi: 10.1016/j.freeradbiomed.2025.03.011 40086491

[37] Button EL Dwyer E Lewis JB Mortensen MS McDonald E Butler E . The 1 -cys peroxiredoxin, PRDX-6, suppresses an NHR-49-dependent pro-survival response, including the flavin monooxygenase, FMO-2, that protects against fungal and bacterial infection. Redox Biol. (2025) 91:103992. doi: 10.1016/j.redox.2025.103992 41702017 PMC12930036

[38] Jia W Wang C Zheng J Li Y Yang C Wan QL . Pioglitazone hydrochloride extends the lifespan of caenorhabditis elegans by activating DAF-16/FOXO- and SKN-1/NRF2-related signaling pathways. Oxid Med Cell Longevity. (2022) 2022:8496063. doi: 10.1155/2022/8496063 35677109 PMC9168093

[39] Liu F Wang H Zhu X Jiang N Pan F Song C . Sanguinarine promotes healthspan and innate immunity through a conserved mechanism of ROS-mediated PMK-1/SKN-1 activation. iScience. (2022) 25:103874. doi: 10.1016/j.isci.2022.103874 35243236 PMC8857505

[40] Zhu X Liu F Wu Q Li S Ruan G Yang J . Brevilin a enhances innate immunity and the resistance of oxidative stress in caenorhabditis elegans via p38 MAPK pathway. Int Immunopharmacol. (2022) 113:109385. doi: 10.1016/j.intimp.2022.109385 36330917

[41] Xiao Y Zhang L Liu Y . Protocol for assessing the healthspan of caenorhabditis elegans after potential anti-aging drug treatment. STAR Protoc. (2023) 4:102285. doi: 10.1016/j.xpro.2023.102285 37148246 PMC10193290

[42] Xiao Y Zhang H Sheng Y Liu F Gao J Liu G . Usnic acid extends healthspan and improves the neurodegeneration diseases via mTOR/PHA-4 signaling pathway in caenorhabditis elegans. iScience. (2022) 25:105539. doi: 10.1016/j.isci.2022.105539 36425761 PMC9679492

[43] Xiao Y Liu F Kong Q Zhu X Wang H Li S . Metformin induces S-adenosylmethionine restriction to extend the caenorhabditis elegans healthspan through H3K4me3 modifiers. Aging Cell. (2022) 21:e13567. doi: 10.1111/acel.13567 35146893 PMC8920454

[44] Calahorro F Holden-Dye L O’Connor V . Impact of drug solvents on C. elegans pharyngeal pumping. Toxicol Rep. (2021) 8:1240–7. doi: 10.1016/j.toxrep.2021.06.007 34195015 PMC8233170

[45] Xiao Y Liu F Zhao PJ Zou CG Zhang KQ . PKA/KIN-1 mediates innate immune responses to bacterial pathogens in caenorhabditis elegans. Innate Immun. (2017) 23:656–66. doi: 10.1177/1753425917732822 28958206

[46] Xiao Y Han C Li X Zhu X Li S Jiang N . S-adenosylmethionine (SAM) diet promotes innate immunity via histone H3K4me3 complex. Int Immunopharmacol. (2024) 131:111837. doi: 10.1016/j.intimp.2024.111837 38471365

[47] Zhou H Shen Y Dong C Feng W Tian Y Xiao Y . Asperuloside promotes innate immunity via IRE-1/XBP-1 mediated unfolded protein response. Bioorg Chem. (2025) 157:108318. doi: 10.1016/j.bioorg.2025.108318 40024200

[48] Xiong L Pereira De Sa N Zarnowski R Huang MY Mota Fernandes C Lanni F . Biofilm-associated metabolism via ERG251 in candida albicans. PloS Pathog. (2024) 20:e1012225. doi: 10.1371/journal.ppat.1012225 38739655 PMC11115363

[49] Rebai Y Wagner L Gnaien M Hammer ML Kapitan M Niemiec MJ . Escherichia coli nissle 1917 antagonizes candida albicans growth and protects intestinal cells from C. albicans-mediated damage. Microorganisms. (2023) 11:1929. doi: 10.3390/microorganisms11081929 37630490 PMC10457924

[50] Cui Y Wang R Li X Bai G Xiao Y . Ginkgolide a enhances the resistance to pathogen infection through mitochondrial unfolded protein response. Cell Mol Life Sci: CMLS. (2025) 82:349. doi: 10.1007/s00018-025-05869-5 41055707 PMC12504166

[51] Liu F Wang Q Xiong J Wang M Zhou H Xiao Y . Parental S-adenosylmethionine diet defines offspring immune response via histone H3K4me3 complex and endoplasmic reticulum UPR. Cell Commun Signaling. (2025) 23:397–409. doi: 10.1186/s12964-025-02386-7 40993686 PMC12462273

[52] Xiao Y Hong CA Liu F Shi D Zhu X Yu C . Caffeic acid activates mitochondrial UPR to resist pathogen infection in caenorhabditis elegans via the transcription factor ATFS-1. Infect Immun. (2024) 92:e00494-23. doi: 10.1128/iai.00494-23 38294242 PMC10929418

[53] Xiao Y Li L Han C Huang T Ren S Wang X . Chlorogenic acid inhibits pseudomonas toxin pyocyanin and activates mitochondrial UPR to protect host against pathogen infection. Sci Rep. (2025) 15:5508. doi: 10.1038/s41598-025-90255-1 39953205 PMC11829045

[54] Sun J Singh V Kajino-Sakamoto R Aballay A . Neuronal GPCR controls innate immunity by regulating noncanonical unfolded protein response genes. Science. (2011) 332:729–32. doi: 10.1126/science.1203411 21474712 PMC3125668

[55] Fan Z Zhang H Liu F Xiao Y . Parental sodium benzoate diet induces fat accumulation in offspring via histone H3K9me3 and SKN-1/Nrf2. Curr Res Food Sci. (2026) 12:101299. doi: 10.1016/j.crfs.2025.101299 41551184 PMC12808894

[56] Liu F Xiao Y Ji XL Zhang KQ Zou CG . The cAMP-PKA pathway-mediated fat mobilization is required for cold tolerance in C. elegans. Sci Rep. (2017) 7:638–47. doi: 10.1038/s41598-017-00630-w 28377576 PMC5428847

[57] Xiao Y Cui Y Zhang Y Fu W Liu Y Liu F . Berberine hydrochloride enhances innate immunity to protect against pathogen infection via p38 MAPK pathway. Front Immunol. (2025) 16:1536143. doi: 10.3389/fimmu.2025.1536143 40092994 PMC11906452

